# Plant chemical defence: a partner control mechanism stabilising plant - seed-eating pollinator mutualisms

**DOI:** 10.1186/1471-2148-9-261

**Published:** 2009-11-03

**Authors:** Sébastien Ibanez, Christiane Gallet, Fanny Dommanget, Laurence Després

**Affiliations:** 1 Laboratoire d'Ecologie Alpine UMR CNRS 5553 Université Joseph Fourier B.P.53, 38041 Grenoble CEDEX 9 France; 2 Laboratoire d'Ecologie Alpine UMR CNRS 5553 Université de Savoie F-73376, Le Bourget-du-lac, France; 3 Station Alpine Joseph Fourier UMS CNRS 2925 Université J. Fourier, BP 53, F-38041 Grenoble, France

## Abstract

**Background:**

Mutualisms are inherently conflictual as one partner always benefits from reducing the costs imposed by the other. Despite the widespread recognition that mutualisms are essentially reciprocal exploitation, there are few documented examples of traits that limit the costs of mutualism. In plant/seed-eating pollinator interactions the only mechanisms reported so far are those specific to one particular system, such as the selective abortion of over-exploited fruits.

**Results:**

This study shows that plant chemical defence against developing larvae constitutes another partner sanction mechanism in nursery mutualisms. It documents the chemical defence used by globeflower *Trollius europaeus *L. (Ranunculaceae) against the seed-eating larvae of six pollinating species of the genus *Chiastocheta *Pokorny (Anthomyiidae). The correlative field study carried out shows that the severity of damage caused by *Chiastocheta *larvae to globeflower fruits is linked to the accumulation in the carpel walls of a C-glycosyl-flavone (adonivernith), which reduces the larval seed predation ability per damaged carpel. The different *Chiastocheta *species do not exploit the fruit in the same way and their interaction with the plant chemical defence is variable, both in terms of induction intensity and larval sensitivity to adonivernith.

**Conclusion:**

Adonivernith accumulation and larval predation intensity appear to be both the reciprocal cause and effect. Adonivernith not only constitutes an effective chemical means of partner control, but may also play a key role in the sympatric diversification of the *Chiastocheta *genus.

## Background

Conflicts of interest are frequent in interspecific mutualisms [1,2]. Plant/seed-eating pollinator mutualisms involve a plant pollinated by an insect whose larvae develop upon the plant's seeds. In these nursery pollination mutualisms, the conflict lies in the number of seeds eaten by the pollinator's larvae that therefore will not contribute to the plant's fitness [3-6]. As a consequence, evolutionary theory predicts that plants evolve traits that limit the costs imposed by the insect partners. Despite this broad prediction, attempts to identify mechanisms of partner control in nursery mutualisms have so far fell short in pinpointing a general mechanism. Pellmyr & Huth [7] showed that the selective abortion of fruits in the *Yucca *- *Yucca *moth interaction was an effective defence against the developing larvae, but this mechanism was found in only one of the three *Yucca *- *Yucca *moth systems studied by Adicott & Bao [8]. Selective abortion may not provide a general explanation for the stability of this mutualism [9]. Instead, density-dependent mortality in oviposition-induced 'damage zones', a characteristic specific to this system, may be a more important mechanism in terms of the regulation of the interaction [10]. Selective fruit abortion is also part of the *Silene latifolia*-*Hadena bicruris *interaction [11,12]. Holland's investigation of the *Senita *cactus - *Senita *moth system [13] found no evidence of selective abortion but suggested that excess flower production followed by massive fruit abortion might actually increase a plant's male fitness, rather than serving to control seed predation by pollinator larvae. In the fig-fig wasp system it is theoretically possible that several mechanisms for reducing the plant's costs coexist [14]. The geometry of the fig seems to play a crucial role in limiting the intensity of the damage inflicted by wasp larvae. Indeed, fig wasps preferentially oviposit in the inner ovules and avoid the outer ovules [15] presumably because the wasp larvae which develop in the outer ovules are more exposed to parasitoids that oviposit from outside the syconia than the larvae developing in the inner ovules [16]. The use by plants of chemicals to kill non-mutualistic pests or limit the damage they cause is a very common phenomenon [17,18] which may also play a role in mutualistic interactions. So far however, the importance of induced plant chemical defence in partner control has not been explored.

**Figure 1:**
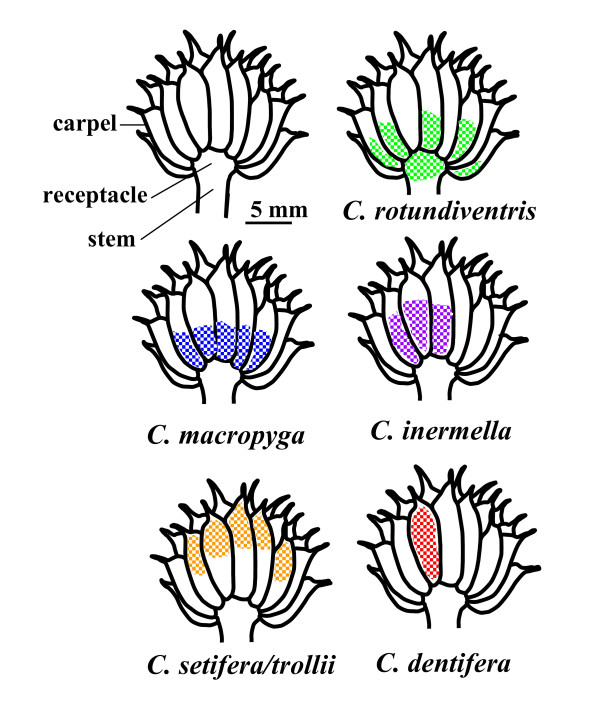
**Exploitation patterns**. Fruit architecture and the exploitation pattern of a single larva for each species studied. Exploitation patterns. Fruit architecture and the exploitation pattern of a single larva for each species studied.

Here we studied the interaction between the European globeflower *Trollius europaeus *(L.) and its seed-eating pollinators *Chiastocheta spp*. (Pokorny) and tested whether plants can limit seed predation through chemical defence. The European globeflower *Trollius europaeus *L. (Ranunculaceae) is an arctic-alpine perennial species growing in moist meadows. Each individual typically produces a single yellow flower composed of around 10 tightly-closed sepals which form a globose corolla that contains approximately 10 nectariferous staminodias, 30 multiovulate carpels, and numerous stamens that sequentially dehisce throughout flower longevity (typically 5-9 days [19,20]). In the Alps, the plant is passively pollinated by six species of *Chiastocheta *flies (Anthomyiidae): *C. rotundiventris *Hennig, *C. dentifera *Hennig, *C. inermella *Zetterstedt, *C. macropyga *Hennig, *C. setifera *Hennig, and *C. trollii *Zetterstedt. *Chiastocheta *flies are the only pollinators of *T. europaeus *and *Chiastocheta *larvae feed only on *T. europaeus *seeds [21,22]. The female deposits one or several eggs on, or between the carpels, at various flower stages depending on the species [19,21]. Shift in oviposition time among species ranges from 2 days to one week [23]. Egg morphology, colour and position on the fruit make it possible to assign them to a species [24], except for *C. trollii *and *C. setifera *that cannot be distinguished based on egg features. The early ovipositing fly species *C. rotundiventris *visits young, unpollinated flowers, and typically deposits just one egg per flower. The late ovipositing species *C. dentifera *lays several eggs on pollinated, fading flowers. After hatching, larvae develop on seeds throughout fruit maturation (about 4 weeks). Larvae from each species have a specific location in the globeflower complex fruit, composed of several follicles (hereafter referred to as carpels, Figure 1). The larva of the early ovipositing species *C. rotundiventris *is found in the floral receptacle; it enters several carpels successively through their bases and eats one to several seeds in each carpel, so that many carpels are damaged by this species. In contrast, the larva of the late ovipositing species *C. dentifera *is found in one single carpel and consumes most of its seeds, thereby inflicting only limited damage to the carpels. The larvae of the intermediate species forage their way through several carpels inflicting various levels of damage to carpels (Figure 1). At the end of their development, the larvae exit the fruit and drop into the soil to overwinter as pupae.

The globeflower cannot respond to over-exploitation by *Chiastocheta *larvae with the selective abortion of parasitized fruits, as it only produces one to three flowers per blooming, whereas yucca and senita cactus produce hundreds of flowers. Nor is the selective abortion of parasitized carpels an option because developing larvae move freely from one carpel to another.

We hypothesize that the accumulation in the globeflowers carpel walls of a luteolin based flavonoid, adonivernith (luteolin 8-β-d-glucopyranosyl-2"-*O*-d-xylopyranoside) with increasing number of developing larvae in the fruit [25] is a mechanism of partner control. Indeed, the unparasitized fruits (artificially protected from ovipositing flies) contain significantly lower amounts of adonivernith than the parasitized fruits, suggesting that this compound is induced by larvae infestation and will act as a defence compound. Unfortunately, as *Chiastocheta *larvae cannot be reared on an artificial medium, this hypothesis could not be confirmed by means of *in vitro *toxicity experiments. However, other flavonoids have been identified as active inhibitors of larval growth on the larvae of the corn earworm (*Heliothis zea *[26], the autumnal moth *Epirrita autumnata *[27], and the fall armyworm *Spodoptera frugiperda *[28,29]). In other cases, flavonoids have been described as feeding deterrents against the American butterfly *Pieris napi oleracea *[30], the stink bug *Nezara viridula *[31], the storage pest *Sitophilus zeamais *[32], and the mustard leaf beetle *Phaedon cochleariae *[33]

We hypothesise that adonivernith, the most abundant phenolic compound found in the carpel walls of *T. europaeus*, constitutes a chemical plant defence against *Chiastocheta *larvae by acting as a larval growth inhibitor or as a feeding deterrent. We predict that the accumulation of adonivernith in the carpel walls following larval damage will limit seed predation per damaged carpel. Moreover, several species of *Chiastocheta *coexist in *T. europaeus *populations. They all feed on globeflower's seeds (which do not contain adonivernith [25]), but differ in terms of their exploitation pattern inside the fruit [23] and in the level of damage inflicted to carpels. Each *Chiastocheta *species may induce and react to adonivernith in a specific way.

In order to test whether adonivernith induction is a means for globeflowers to control seed predation by pollinators' larvae, we carried out a field study on *T. europaeus *flowers in which we left only one egg of one of the different *Chiastocheta *species present (Figure 1). We dissected the fruit after full larval development and measured the mass of the larva, the number of damaged carpels, and the number of seeds eaten. We also estimated the fruit's seed/ovule ratio and the concentration of adonivernith in the carpel walls. More specifically we asked the following questions: Is adonivernith concentration correlated with the level of larval damage to the plant? Is adonivernith concentration correlated with larval mass? Is adonivernith concentration correlated with the number of seeds eaten? How do the *Chiastocheta *species differ in terms of adonivernith induction? Is the plant's chemical defence as efficient to control the different *Chiastocheta *species?

## Results

### Adonivernith concentration and the intensity of larval damage

**Figure 2:**
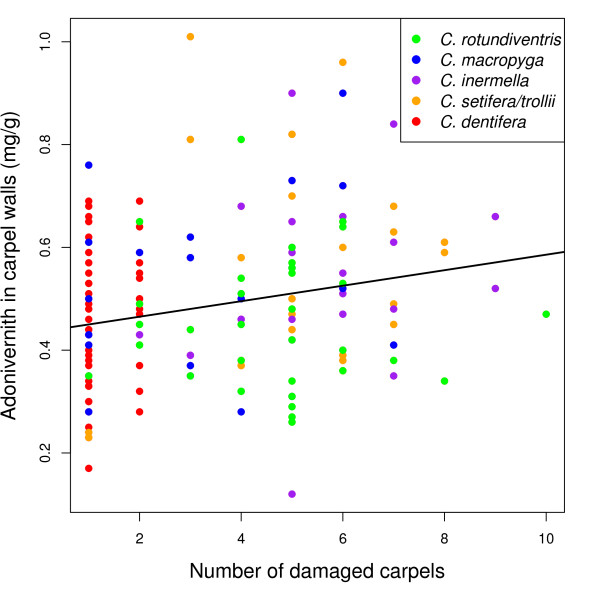
**Adonivernith induction**. Adonivernith concentration in the carpel walls according to the number of damaged carpels. Green: *C. rotundiventris*, blue: *C. macropyga*, purple: *C. inermella*, orange: *C. setifera/trollii*, red: *C. dentifera*. See Table 2 for the statistical significance of the relationship.

The variation in adonivernith concentration between individual plants was wide enough to carry out the statistical analysis (range 0.12-1.01 mg/g, mean 0.48 mg/g, coefficient of variation 0.32). Adonivernith concentration positively correlated with the number of damaged carpels when considering all species together (Linear Model LM, t_1,152 _= 2.75, p = 0.007, Table 1 & Figure 2). Although not significant due to the small sample size and high variability, the correlation was also positive when the species were analysed separately, except for *C. setifera/trollii *(Table 1). The seed/ovule ratio was not dependent on the number of damaged carpels (Generalised Linear Model GLM, binomial family, t_1,152 _= 0.107, p = 0.91).

**Table 1:** Adonivernith induction

	**Regression coefficient**	**t**	**p-value**	**Residuals d.f**.	**R^2^**
All species	0.02	2.75	0.007	152	0.05
*C. rotudiventris*	0.02	0.87	0.39	22	0.03
*C. macropyga*	0.02	1.24	0.23	23	0.06
*C. inermella*	0.02	0.98	0.34	20	0.05
*C. setifera/trollii*	-0.002	-0.13	0.90	32	0.001
*C. dentifera*	0.01	0.40	0.69	47	0.003

Adonivernith concentration in carpel walls in relation to the number of damaged carpels (univariate linear model).

### Larval traits and adonivernith concentration

**Figure 3:**
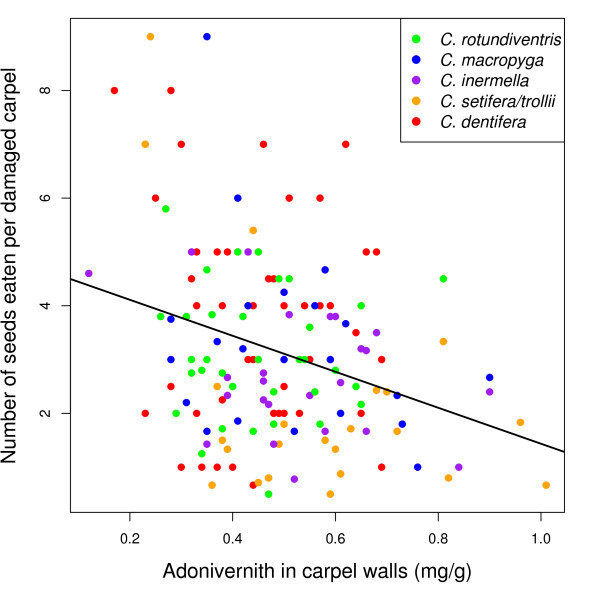
**Adonivernith effect**. Number of seeds eaten per damaged carpel according to the adonivernith concentration in the carpel walls. Green: *C. rotundiventris*, blue: *C. macropyga*, purple: *C. inermella*, orange: *C. setifera/trollii*, red: *C. dentifera*. See Table 3 for the statistical significance of the relationship.

There was no link between larval mass and adonivernith concentration in the carpel walls when the species were pooled (ANOVA, F_1,137 _= 0.0044, p = 0.94) nor when they were analysed separately (not shown, p > 0.12 in all cases). Similarly, there was no link between the *total *number of seeds eaten per larva and adonivernith concentration when the species were pooled (ANOVA, F_1,152 _= 0.0018, p = 0.96) nor when they were analysed separately (not shown, p > 0.51 in all cases). However, when the number of seeds eaten *per damaged carpel *was considered, adonivernith had a negative effect (LM, t_1,151 _= -4.44, p < 1E-4, Table 2 & Figure 3), and the seed/ovule ratio a positive effect on seed predation (LM, t_1,151 _= 5.58, p < 1E-6, Table 2) when the species were pooled. The R^2 ^of the corresponding multivariate linear model was 0.25 (when the species were treated separately, the R^2 ^were between 0.11 and 0.40, Table 2). Larval mass positively correlated with the number of seeds eaten per larva when all the species were pooled (t_1,137 _= 6, p < 1E-7, Table 3). When the species were analysed separately, the link was significant for *C. rotudinventris, C. inermella *and *C. dentifera *(Table 3).

**Table 2:** Seed predation

	**Adonivernith effect**			**Seed/ovule ratio effect**			**Model**	
	**Regression coefficient**	**t**	**p-value**	**Regression coefficient**	**T**	**p-value**	**Residuals d.f**.	**R^2^**
All species	-3.46	-4.44	<1E-4	3.51	5.58	<1E-6	151	0.25
*C. rotudiventris*	-4.27	-2.22	0.04	2.73	1.43	0.17	21	0.27
*C. macropyga*	-2.42	-1.69	0.10	0.52	0.40	0.69	22	0.12
*C. inermella*	-4.66	-2.47	0.02	5.51	3.02	0.01	19	0.40
*C. setifera/trollii*	-0.71	-0.44	0.66	2.57	1.92	0.06	31	0.11
*C. dentifera*	-2.05	-1.20	0.24	5.41	5.40	<1E-5	46	0.40

Number of seeds eaten per damaged carpel in relation to adonivernith concentration in the carpel walls and the developing seed/ovule ratio (multivariate linear model).

**Table 3:** Larval growth

	**Regression coefficient**	**t**	**p-value**	**Residuals d.f**.	**R^2^**
All species	0.07	6.00	<1E-7	137	0.21
*C. rotudiventris*	0.12	4.28	<1E-3	22	0.45
*C. macropyga*	-0.001	-0.03	0.98	20	<1E-4
*C. inermella*	0.12	3.43	0.003	19	0.38
*C. setifera/trollii*	0.05	1.26	0.22	28	0.05
*C. dentifera*	0.19	4.44	<1E-4	40	0.33

Larval mass in relation to the number of seeds eaten (univariate linear model).

### Differences between *Chiastocheta *species

**Figure 4:**
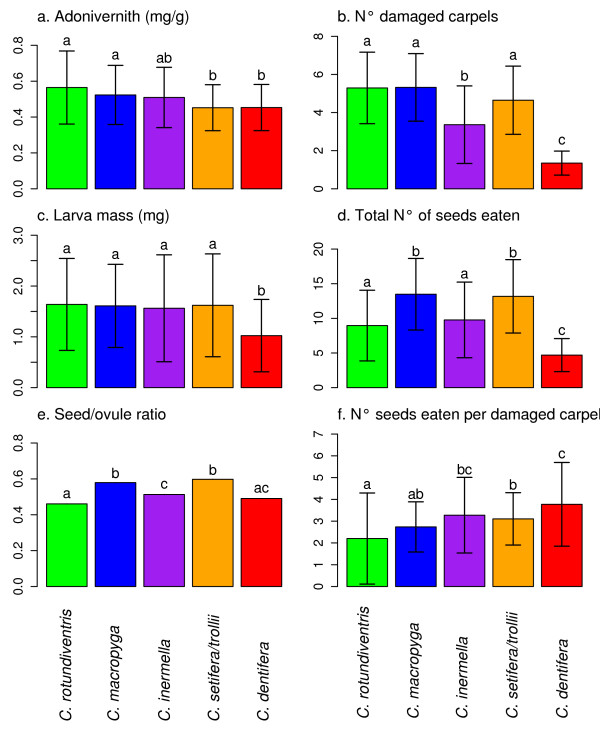
**Differences between fruits infested by different *Chiastocheta *species**. Mean (bar) and standard deviation (bracket) for each group of fruits infested by the different *Chiastocheta *species of: a. adonivernith concentration in carpel walls in mg/g, b. number of damaged carpels by a single larva, c. larval mass in mg, d. total number of seeds eaten per larva, e. seed/ovule ratio and f. number of seeds eaten per damaged carpel.

The adonivernith concentrations differed between fruits infested by different species. Fruits infested by *C. rotudiventris *and *C. macropyga *larvae had higher concentrations than those infected by *C. setifera/trollii *and *C. dentifera *larvae (ANOVA, Figure 4.a). The number of damaged carpels differed between species, *C. rotudiventris *damaged the most carpels, closely followed by *C. macropyga *and *C. setifera/trollii*. *C. inermella *damaged around 3 carpels whereas *C. dentifera *damaged no more than two carpels (Figure 4.b). Larval mass differed between species;*C. dentifera *was the smallest species (Figure 4.c). The total number of seeds eaten per larva varied across species: *C. macropyga *and *C. setifera/trollii *ate more seeds than the others, followed by *C. rotudiventris *and *C. inermella*, and then *C. dentifera *(Figure 4.d). The seed/ovule ratio differed between species: *C. macropyga *and *C. setifera/trollii *had the highest ratio and *C. rotudiventris *the lowest (Figure 4.e). *C. dentifera *ate the most seeds per damaged carpel, and *C. rotudiventris *the least (Figure 4.f).

## Discussion

### Advantages and disadvantages of a correlative study

In a previous study, Gallet *et al *[25] showed that the amount of adonivernith in the carpel walls positively correlated to the number of larvae in the fruit. Here we only consider fruits infested by a single larva and show that 1) adonivernith concentration is dependent on the amount of damage (the number of damaged carpels) caused by the larva and 2) the number of seeds eaten per damaged carpel decreases as adonivernith concentration increases. *Chiastocheta *larvae are specific to *Trollius *fruits, and cannot be reared on artificial medium under controlled laboratory conditions. Therefore we could not directly carry out bioassays to show that the cause of the reduced seed consumption is indeed adonivernith. Other correlated factors may be involved in the plant's response to larval damage and in its toxicity against larvae. For example, Gallet *et al *[25] showed that other undetermined phenolic compounds respond to increasing numbers of larvae, although the response is more marked with adonivernith. The chemical defence probably involves several compounds and possible synergistic effects: some may be precursors or the degraded compounds of others, and some may be more toxic to the larvae than others. Only bioassays performed in controlled conditions can link a cause (adonivernith concentration) to an effect (larval mass), but the correlative field study has the advantage of showing that the phenomenon is indeed at work in nature [34]. The huge variability of flavonoids in the natural environment [35-37], coupled with the wide range of factors that may influence their production and accumulation means an *in vitro *experiment would be entirely disconnected from nature and is unrealistic. Seed-eating pollinator mutualisms are complex systems to which observational studies or semi-experimental field studies are better adapted [7,11,16].

### Disentangling cause and effect

**Figure 5:**
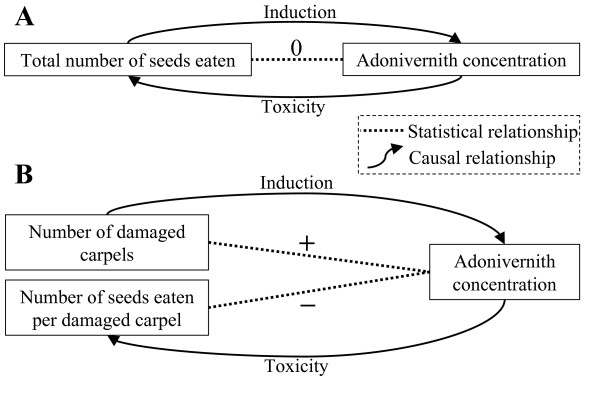
**Disentangling cause and effect**. Statistical and causal relationships at play in the system. A. The total number of seeds eaten is simultaneously cause and effect of adonivernith concentration. B. The number of damaged carpels is the cause of the induction of defence, while the number of seeds eaten per damaged carpel is the effect of defence. Dotted line: statistical relationship. Arrow: causal relationship.

Another advantage of the correlative approach is that it makes it possible to disentangle two processes which come into play simultaneously: the induction of plant defence (in response to carpel damage inflicted by larval predation) and the consequences of defence induction (on larval predation). The plant defence and larval predation are both the cause and effect of adonivernith induction [38]. This explains why no direct link was found between adonivernith concentration and the total number of seeds eaten, nor between adonivernith concentration and larval mass: more seeds eaten means more carpel damage and therefore more adonivernith induction, but at the same time more adonivernith induction means less seeds eaten (Figure 5.a). Instead, adonivernith induction can be explored by looking at the link between adonivernith concentration and the number of damaged carpels, and toxicity can be measured in terms of the link between the number of seeds eaten per damaged carpel and adonivernith concentration (Figure 5.b).

Plant reactions to the damage vary between individuals, leading to variations in adonivernith concentrations in fruits with the same amount of damage. Thanks to this natural variability of plant defences, we were able to show that in the most reactive fruits, the larvae ate less seeds per damaged carpel. The variability of plant defences can have a genetic (*e.g*. [39]) or an environmental (*e.g*. [40]) basis.

### Origin of the chemical defence

Adonivernith is abundant in almost all parts of the globeflower, especially in the leaves and sepals [25]. It is probably involved in the defence against herbivores and florivores, as well as in the resistance to ultra-violet radiation. Ultra-violet radiation has been shown to induce adonivernith production in globeflowers (S. Ibanez, unpublished results), and globeflower populations located at high altitudes contain higher concentrations of adonivernith in their carpel walls [25]. Adonivernith was first described in the genus *Adonis *[41], the sister genus of *Trollius *[42]. It is also present in other *Trollius *species [25], which suggests that it was already present in the common ancestor of *Trollius *and *Adonis*. The chemical defence used by *T. europaeus *against *Chiastocheta *larvae is probably an exaptation. However, the accumulation of adonivernith in carpel walls is not induced by mechanical damage and appears to be specifically induced by *Chiastocheta *larvae [25].

### The ecological and evolutionary stability of the interaction

When several larvae are allowed to develop in a single fruit, each larva is exposed to increasing amounts of adonivernith as the number of larvae developing in the fruit increases [25]. The mechanism is therefore density-dependant: the higher the population density of *Chiastocheta*, the more it suffers from chemical defence. The density-dependant mechanism is also found in yuccas [10]. In two models exploring the evolutionary emergence of fruit abortion in yucca and senita cactus [43,44], Holland *et al *show that density-dependant mechanisms which limit seed predation by moths can maintain the costs of seed predation at a lower level than the benefits of pollination thereby stabilising the interaction. This ensures the ecological stability of the interaction in the sense that globeflower populations are more likely to persist. The modelling carried out by Ferdy *et al *[45] showed that if the closure of the globeflower corolla led to an increase in intraspecific contest competition due to an increase in egg survival, then females would evolve a reduced clutch size per flower thus stabilising the interaction, but unpublished field data (L. Després) does not support the model hypothesis (i.e. higher egg survival in closed corolla). However, the chemical defence mechanism described here may play exactly the same role as globe closure in Ferdy et al's model if it indirectly increases intraspecific competition between larvae. The chemical defence would then lead to an evolutionary stabilisation of the mutualism. Finally, the larvae are likely to evolve a resistance to adonivernith. Preliminary results suggest that the activity of the detoxifying enzyme cytochrome P450 (frequently involved in insect resistance to plant chemicals [46]) in *Chiastocheta *larvae is greater when they are exposed to adonivernith (L. Després, unpublished results). In any case the results of this study suggest that adonivernith is more likely to act as a growth inhibitor or a feeding deterrent rather than a lethal compound.

### Plant defence and sympatric speciation in the *Chiastocheta *genus

Phylogenetical and biogeographical data indicate that the diversification of the *Chiastocheta *genus mostly occurred in sympatry [47]. The dominance of intra- over inter-specific competition could have driven the radiation [48] through resource partitioning in space (exploitation pattern [23]) and time (oviposition time [48]). Both processes are affected by plant defence: exposure to adonivernith will depend on the exploitation pattern, and the larvae of late-ovipositing species will be exposed to higher concentrations resulting from the damage inflicted by early-ovipositing species. The accumulation of adonivernith in the carpel walls will depend on the exploitation pattern (the number of damaged carpels) and on oviposition timing. Interestingly, the larva of the late-ovipositing species *C. dentifera *only mines through a single carpel, thereby avoiding contact with the carpel walls containing adonivernith. In the present study, it is the species which least induces a plant's response, and the least sensitive to adonivernith. Intra- and inter-specific competition may be direct in the form of larval contests and the data presented here suggests that it may also be indirect by means of adonivernith induction. Adonivernith may have played a key role in the sympatric speciation of the *Chiastocheta *genus through the following three mechanisms: 1) by increasing competition between larvae; 2) by provoking a behavioural avoidance strategy in *C. dentifera*; and 3) by mobilising different capacities to metabolize this chemical compound. We have already shown that larval foraging behaviour varies across species and we predict that the larval capacity for resistance also varies across species.

## Conclusion

Adonivernith induction by larvae and adonivernith toxicity on larvae are two interlinked processes: adonivernith accumulation and larval predation are both the reciprocal cause and effect. The interaction between the larvae and adonivernith varies between the six *Chiastocheta *species, which may have played a role in the sympatric speciation of the genus. Adonivernith induction reduces the costs of mutualism for the plant, which has a stabilising effect on the plant's pollination specialisation on *Chiastocheta *flies.

## Methods

### Field study design

We conducted the field study around the "Station Alpine Joseph Fourier UMS 2925", col du Lautaret, France, in a single large population "Ruillas", 2025 m a.s.l in June -July 2007. A sample of 289 flowers was chosen randomly and left untouched until naturally pollinated. We then removed the eggs from each flower and waited one day for a new set of ovipositing females to lay their eggs. At the end of the day, we inspected the flowers and removed all the newly-laid eggs but one. The flower was then covered with a nylon bag to prevent further oviposition. If no eggs were found, we repeated the same procedure the following day. We recorded the day each flower was bagged (ranging from June 8^th ^to 19^th^) and collected them 28 days later.

Back in the laboratory, the fruits were stored at 4°C for a maximum duration of 24 h before dissection. For each fruit, all carpels were checked for damage, and the number of damaged carpels recorded. Five intact carpels were chosen at random and dissected, and the ratio of the number of developing seeds to the number of ovules (developing and degenerating) per carpel was determined. All damaged carpels were dissected in order to estimate the number of developing seeds that had been eaten [3]. The larvae were located either in the damaged carpels, or in the flower receptacle (in the case of *C. rotundiventris*), and weighed. The position and the trajectory of each larva inside the fruit (Figure 1) were used to assign it to one of the five following species: *C. rotundiventris*, *C. macropyga*, *C. inermella*, *C. setifera *or *C. trollii *(recorded as *C. setifera/trollii*, as these two species cannot be distinguished at the egg or larval stage [19]) and *C. dentifera*. If the fruit happened to contain no larvae, or more than one larva, it was excluded from the analysis. The carpel walls of five damaged carpels and the five intact carpels used for pollination analysis were pooled for the chemical analysis as preliminary results had shown that adonivernith concentration in intact carpels as opposed to damaged carpels was not significantly different (F_1,29 _= 2.039, p = 0.164). If the larva had damaged less than five carpels, the intact carpels were chosen at random and dissected so that all chemical analyses were carried out on ten carpel walls. Of the 289 flowers included in the first stage of the design, 154 were used for the statistical analysis. Missing samples were either lost in the field, contained no, or more than one larva (some hidden eggs might have been missed), or had been consumed by herbivores such as bush crickets (Tettigoniidae species) despite the protection offered by the nylon bag. *C. rotundiventris *developed in 24 of the 154 fruits, *C. macropyga *in 25, *C. inermella *in 22, *C. setifera/trollii *in 34 and *C. dentifera *in 49.

### Chemical analysis

All samples were individually stored at -18°C until analysis. This individual storage and the very small size of some of the samples meant dry weight could not be measured: all the results were given as fresh weight (FW). Each sample was weighed and extracted using 50 ml of an ethanol-water (50/50) mixture under reflux [25]. Aliquots (20 μl) of the ethanolic solution were used for HPLC analysis on a RP C18 μBondapak column, 4.6 mm × 250 mm, monitored using a Waters 600 Controller. Spectra were recorded on a Waters 996 PDA. Solvent A was acetic acid 0.5% in distilled water and solvent B acetic acid 0.5% in acetonitrile. Adonivernith was separated with an isocratic flow (1.5 ml min^-1^) of 20% of B in A and its area was recorded at 354 nm. Concentration was expressed in luteolin equivalent, based on a calibration curve established with pure luteolin (obtained from Extrasynthese, Lyon, France).

### Data analysis

All statistical analyses were carried out using the software R 2.6.0 (R Development Core Team 2007). We carried out ANOVAs, univariate and bivariate linear regressions using the R function "lm" in the "stats" package. We produced generalised linear models (binomial family) using the R function "glm" in the "MASS" package. The datasets corresponding to the five taxonomical subdivisions of the *Chiastocheta *genus we used were either analysed all together in order to draw conclusions at the genus level; or analysed separately in order to explore the differences between species.

## Authors' contributions

SI, CG and LD participated in the conception and design of the study. SI and FD carried out the field study and the statistical analysis. CG and SI carried out the chemical analysis. SI, CG and LD drafted the manuscript. All authors have read and approved the final manuscript.
